# Biopsy-Proven Non-glomerular Renal Pathology in Localized Granulomatosis With Polyangiitis Treated With Methotrexate and Avacopan Presenting With New Renal Symptoms: A Case Report

**DOI:** 10.7759/cureus.106031

**Published:** 2026-03-28

**Authors:** Kaandeeban Mohanraj, John Amerson, Naveen Punchayil Narayanankutty

**Affiliations:** 1 Nephrology, Indira Gandhi Medical College and Research Institute, Puducherry, IND; 2 Nephrology, University of South Florida Morsani College of Medicine, Tampa, USA

**Keywords:** acute renal failure, acute tubular necrosis, avocopan, case report, granulomatosis with polyangiitis, methotrexate

## Abstract

Granulomatosis with polyangiitis (GPA) is an antineutrophil cytoplasmic antibody (ANCA)-associated vasculitis that commonly involves the upper respiratory tract and kidneys. The development of new-onset urinary abnormalities, microscopic hematuria, and declining renal function in a patient on treatment with methotrexate and avacopan warrants a prompt renal biopsy to lead management decisions. We describe a case of localized sinonasal GPA in a patient receiving methotrexate and avacopan who presented with new renal symptoms. Laboratory evaluation revealed a serum creatinine of 1.4 mg/dL and a urine protein-creatinine ratio (UPCR) of 0.96 g/g with moderate hematuria and red blood cell casts on urine microscopy. Renal biopsy revealed no evidence of pauci-immune crescentic glomerulonephritis, highlighting the importance of histopathologic confirmation before escalation of immunosuppressive therapy. These findings prevented escalation to stronger immunosuppressive therapy and allowed continuation of the existing regimen. Moreover, the combined use of methotrexate and avacopan in nasal-limited GPA is not well documented, and this case adds more clinical understanding to this therapeutic strategy.

## Introduction

Granulomatosis with polyangiitis (GPA), formerly called Wegener's granulomatosis, is described under the umbrella of antineutrophil cytoplasmic antibody (ANCA)-associated vasculitides. It affects mainly the small- and medium-sized vessels, resulting in pulmonary renal syndrome with additional otolaryngological symptoms [1].

The prevalence in the US has been estimated to be between 120 and 140 cases per million, whereas the annual incidence worldwide is estimated to be between 10 and 20 cases per million people. Clinical manifestations vary greatly, from systemic disease involving multiple organs to localized disease restricted to the upper respiratory tract [1].

In terms of serology, GPA is most frequently linked to anti-proteinase 3 (PR3-ANCA), which is present in about 80% of cases, and anti-myeloperoxidase (MPO-ANCA), which is present in about 15% of cases. A tiny percentage of cases are ANCA-negative. ANCA sensitivity may be lower or negative in patients with localized forms of GPA, but it approaches 90% in patients with generalized disease. The C3 and C4 levels are usually normal, attributed to the non-involvement of the classical pathway [1,2].

Beyond constitutional symptoms, upper respiratory tract involvement is seen in 90% of the affected population at the time of presentation, notably presenting with sinusitis, crusting rhinitis, and saddle nose deformity. Renal involvement is observed only in 10-20% of cases at presentation and eventually in 80% of the patients during the course of the systemic disease, typically manifesting as rapidly progressive crescentic glomerulonephritis [1].

Early identification and treatment are essential for better outcomes. We report a case of nasal-limited GPA presenting with new-onset urinary abnormalities after more than two years of treatment with methotrexate and avacopan therapies.

## Case presentation

A 40-year-old man with a history of nasal-limited GPA, diagnosed in 2023, was admitted in December 2025 for evaluation of persistent microscopic hematuria and long-standing proteinuria. Outpatient nephrology observed an increase in serum creatinine and considerable proteinuria, necessitating a referral for an inpatient renal biopsy.

He noted no constitutional symptoms and denied gross hematuria, dysuria, flank pain, rashes, arthralgia, respiratory symptoms, or recent infections. He reported no exposure to nonsteroidal anti-inflammatory drugs (NSAIDs), drugs, intravenous contrast, or new medications.

The patient first developed thick nasal discharge and crusting along with pain in September 2022. He was evaluated by the otolaryngology team of our hospital, where nasal endoscopy revealed diffuse intranasal crusting and septal perforation.

A nasal septal biopsy revealed significant acute and chronic inflammation, but vasculitis was not detected. He was referred to the rheumatology service, where a diagnosis of localized nasal GPA was made based on the clinical presentation and supporting laboratory results. The patient exceeded the classification threshold of ≥5 points with a cumulative score of 8 points based on the 2022 ACR/EULAR classification criteria for GPA, which includes nasal involvement with crusting and septal perforation (+3) and PR3-ANCA positivity (+5) [3].

Table 1 summarizes important laboratory results at the time of the initial diagnosis.

**Table 1:** Key laboratory findings at the time of initial diagnosis PR3-ANCA: proteinase 3 anti-neutrophil cytoplasmic antibody; MPO-ANCA: myeloperoxidase anti-neutrophil cytoplasmic antibody. Interpretation was based on the 2022 American College of Rheumatology/European Alliance of Associations for Rheumatology classification criteria for granulomatosis with polyangiitis (Robson JC et al., Annals of the Rheumatic Diseases, 2022). © 2022 Author(s). Published by Elsevier. [3]

Laboratory parameter	Value	Reference range
Erythrocyte sedimentation rate (ESR)	34 mm/hr	<20
C-reactive protein (CRP)	23.6 mg/L	<5
Platelet count	410 × 10³/µL	150-400
PR3-ANCA (c-ANCA)	Positive (1.5)	Negative
MPO-ANCA (p-ANCA)	Negative	Negative
Anti-cyclic citrullinated peptide (CCP) antibody	<16 U/mL	<20
Rheumatoid factor	<14 IU/mL	<14
14-3-3 eta protein	<0.2 ng/mL	<0.2
Hepatitis B surface antigen	Negative	Negative
Hepatitis C antibody	Negative	Negative
Urinalysis – protein	1+	Negative

Although urinalysis showed 1+ proteinuria at baseline, renal function was normal, and there were no accompanying urinary sediment abnormalities; therefore, renal involvement was not suspected at the time of diagnosis, and the disease was classified as nasal-limited GPA.

He was started on prednisolone 60 mg, which was later tapered off; methotrexate 15 mg once weekly; avacopan 30 mg bid; folic acid; and trimethoprim-sulfamethoxazole prophylaxis. After starting this treatment, he showed improvement in his condition and achieved clinical remission of GPA in November 2023, while being maintained on injection methotrexate 25 mg subcutaneously once weekly and tablet avacopan 30 mg twice a day.

In light of persistent microscopic hematuria with active urinary sediment and worsening proteinuria, the rheumatology team raised concerns for possible renal involvement of GPA, prompting further nephrologic evaluation.

Along with lumbar and thoracic intervertebral disc degeneration, he also has class 3 severe obesity, hypertension, hyperlipidemia, and obstructive sleep apnea.

There was a family history of kidney disease in the patient’s father and sister; however, the specific diagnosis was unknown to the patient. He quit smoking 10 years ago and denied alcohol or drug use.

On admission, the patient was hemodynamically stable. Vital signs were as follows: blood pressure 140/90 mmHg, pulse rate 96 beats per minute, respiratory rate 15 breaths per minute, temperature 97.6°F, and oxygen saturation 99% on room air. His body mass index (BMI) was 53.25 kg/m². Laboratory studies revealed renal dysfunction with proteinuria and moderate hematuria on urinalysis. Laboratory investigations at admission are summarized in Table 2.

**Table 2:** Laboratory investigations at hospital admission PR3-ANCA: proteinase 3 anti-neutrophil cytoplasmic antibody. Interpretation was based on the KDIGO 2024 Clinical Practice Guideline for ANCA-Associated Vasculitis [4] (open access; CC BY-NC-ND 4.0).

Laboratory parameter	Value	Reference range
Serum creatinine	1.4 mg/dL	0.6–1.2 mg/dL
PR3-ANCA	Negative	Negative
Urinalysis – hematuria	Moderate (2+)	Negative
Urine microscopy	RBC casts present	Absent
Urine protein–creatinine ratio (UPCR)	0.96 g/g	<0.15 g/g

A renal ultrasound demonstrated normal morphology without evidence of obstructive uropathy or mass lesion. During hospitalization, his creatinine improved to 1.1 mg/dL, and he remained clinically stable.

**Figure 1:**
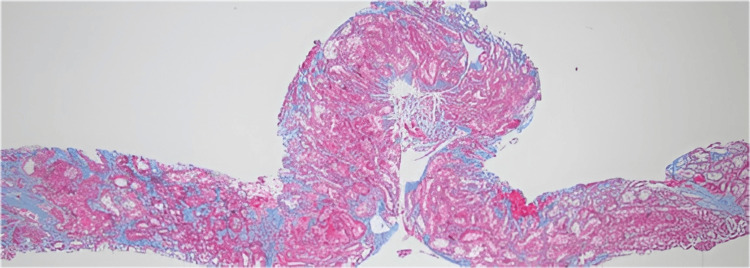
Trichrome stain shows overall mild interstitial fibrosis and tubular atrophy Interpretation was based on the KDIGO 2024 Clinical Practice Guideline for ANCA-Associated Vasculitis [4] (open access; CC BY-NC-ND 4.0).

The renal biopsy demonstrated acute tubular injury with focal cytoplasmic vacuolization, along with superimposed chronic hypertensive nephrosclerosis characterized by mild interstitial fibrosis and tubular atrophy (Figure 1).

**Figure 2:**
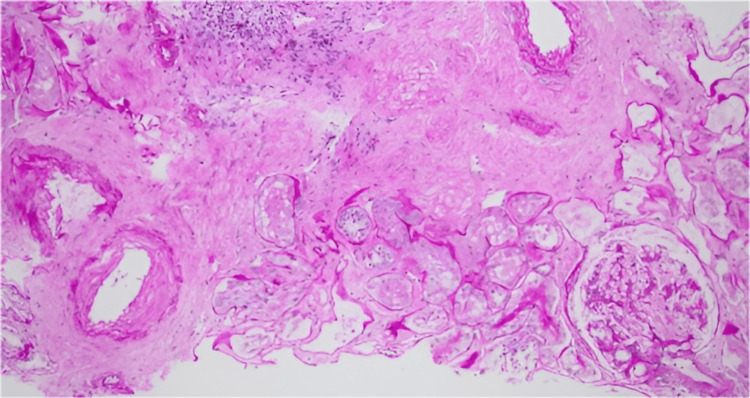
Periodic Acid-Schiff stain shows up to moderate intimal fibrosis in small- to medium-sized arteries Interpretation was based on the KDIGO 2024 Clinical Practice Guideline for ANCA-Associated Vasculitis [4] (open access; CC BY-NC-ND 4.0).

In addition, arteriosclerosis was noted, while crescents, necrotizing lesions, or evidence of vasculitis was not identified (Figure 2).

**Figure 3:**
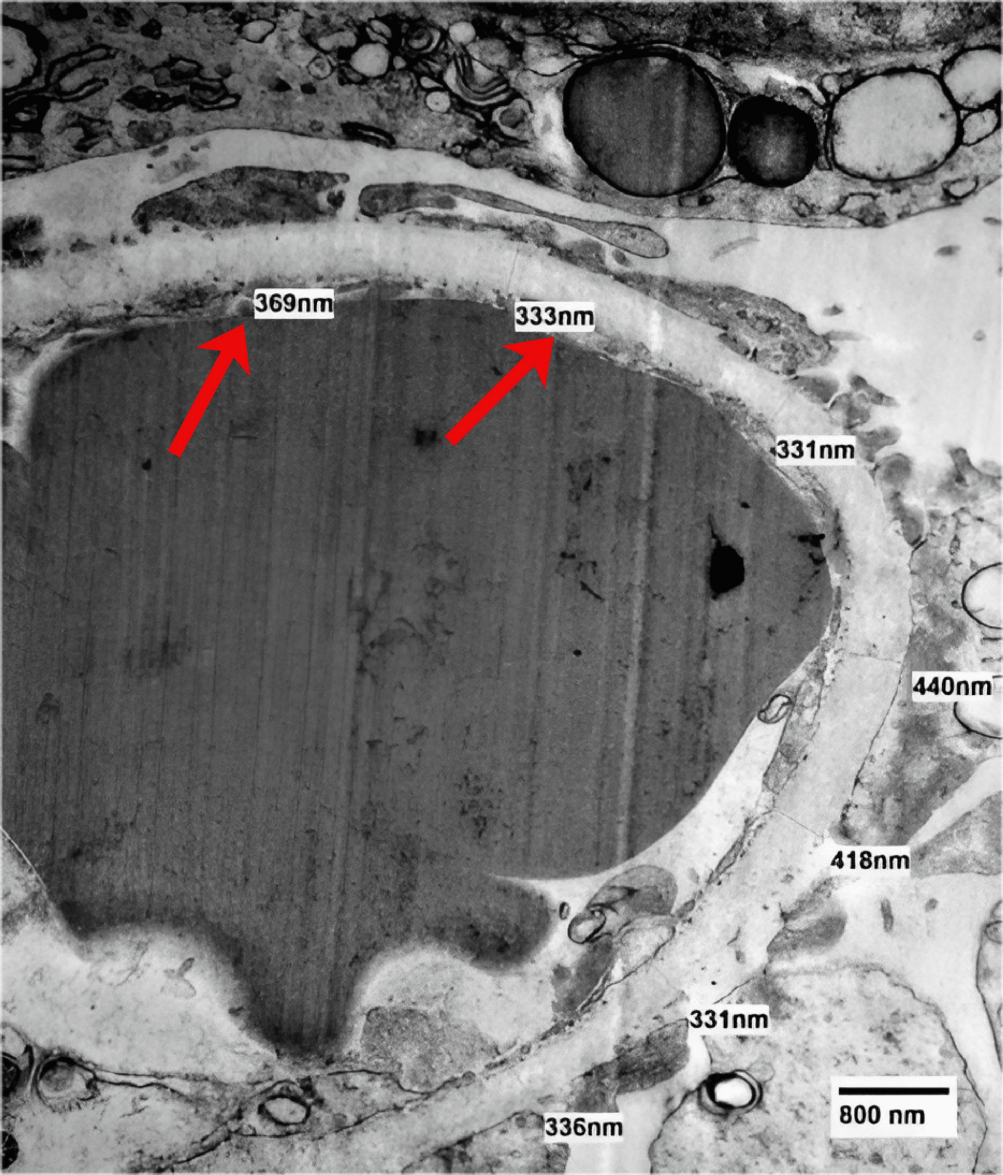
Electron microscopy demonstrating normal glomerular basement membrane thickness without electron-dense immune-complex deposits. Image provided as a representative flat raster image from the diagnostic pathology report. Arrows indicate the glomerular basement membrane (GBM) where thickness measurements were obtained. No electron-dense immune-complex deposits are identified. Interpretation was based on the KDIGO 2024 Clinical Practice Guideline for ANCA-Associated Vasculitis [4] (open access; CC BY-NC-ND 4.0).

In addition, immunofluorescence and electron microscopy were negative for immune complex deposition, effectively ruling out active ANCA-associated glomerulonephritis (Figure 3).

The reliability of the histopathologic findings was supported by the biopsy's 37 glomeruli, which were significantly more than the suggested adequacy threshold (8-10 glomeruli) and the number usually needed to detect focal lesions (>20) [5]. The kidney biopsy findings are summarized in Table 3.

**Table 3:** Summary of key kidney biopsy findings Interpretation was based on the KDIGO 2024 Clinical Practice Guideline for ANCA-Associated Vasculitis [4] (open access; CC BY-NC-ND 4.0).

Domain	Key findings
Overall diagnosis	Acute tubular injury (ATI) with chronic changes consistent with hypertensive nephrosclerosis
Glomeruli	37 glomeruli sampled; 5 globally sclerotic (14%); mild mesangial hypercellularity and matrix expansion; no crescents, necrosis, segmental sclerosis, or endocapillary proliferation
Tubulointerstitium	Diffuse mild ATI with loss of brush borders and focal cytoplasmic vacuolization; mild interstitial fibrosis and tubular atrophy (10–20% cortex); scattered hyaline casts; rare mild tubulitis
Vessels	Mild-moderate arteriosclerosis with arterial intimal fibrosis; mild arteriolar hyalinosis; no vasculitis or endothelialitis
Immunofluorescence	Trace to 1+ smudgy mesangial IgG and light chain staining; 3+ tubular cast staining for IgA, kappa, lambda; C3 (2+) in subset of arterioles/TBMs; no significant immune-complex glomerular deposition
Electron microscopy	Normal GBM thickness; mildly increased mesangial matrix; no electron-dense immune-type deposits; focal podocyte foot process effacement (~20–30%)
Key negative findings	No active necrotizing or crescentic glomerulonephritis, no granulomatous inflammation, no immune-complex or paraprotein deposition

He experienced post-procedural back pain for which he received analgesics. His blood pressure was monitored and managed with lisinopril. He was advised on the continuation of home CPAP and statin therapy for his existing conditions.

He was discharged home in stable condition with plans for nephrology follow-up in two weeks.

## Discussion

Despite the presence of red blood cell casts on urinary microscopy, the kidney biopsy demonstrated no evidence of glomerulonephritis and instead revealed acute tubular injury with chronic hypertensive nephrosclerosis. As urinary sediment findings are not entirely specific, renal biopsy remains the definitive tool for distinguishing glomerular from non-glomerular causes of kidney injury [6,7].

The acute tubular injury observed in this patient was most likely multifactorial, related to chronic hypertensive nephrosclerosis with underlying vascular changes predisposing to ischemic tubular injury, with a possible additional contribution from obesity-associated renal hemodynamic stress.

Minor mesangial changes and focal podocyte foot process effacement were interpreted as nonspecific findings within the overall clinicopathologic context.

GPA predominantly affects the age groups of 40 to 55 years. Several environmental triggers, such as chronic Staphylococcus aureus colonization, silica, hydrocarbons, and certain drug exposures, have been reported to be associated with the pathogenesis. In addition, genetic susceptibility has also been described to play a contributory role, with variants in HLA-DP, SERPINA1, and PRTN3 identified as risk factors, as described by Lyons et al. [1,8].

Recent evidence shows that the pathophysiology mainly dwells in the activation of the alternative complement pathway through C5a. Inflammatory triggers cause proteinase 3 and myeloperoxidase to be presented on the neutrophil cytoplasmic membrane, where ANCA binding leads to neutrophil activation and the release of degradative enzymes. The resulting C5a further amplifies this loop [1,4,9].

The management of GPA is largely determined by disease severity, particularly the involvement of vital organs such as the lungs (alveolar hemorrhage), kidneys (glomerulonephritis), central nervous system, or heart. Our patient is categorized as having a nasal-limited GPA, given the absence of systemic involvement [10].

Localized or nasal-limited GPA, reportedly seen in almost all cases, represents a recognized subset of the disease where the manifestations are predominantly in the upper respiratory tract without systemic involvement [1,3].

Other organs where localized GPA has been reported include the lungs, eyes, and skin. Importantly, the clinical features may overlap with differentials such as allergic and infectious sinusitis, leading to a delay in recognition of the condition [10,11].

Despite being on treatment for over two years with methotrexate and avacopan, our patient developed new-onset microscopic hematuria, proteinuria, and red blood cell casts. The emergence of this constellation of findings raised strong concern for new renal involvement due to relapse. This underscores a teaching point: new urinary abnormalities must undergo cautious interpretation and kidney biopsy, even in the context of apparent clinical remission.

Also, a recent negative PR3 ANCA and the absence of organ-threatening involvement mean that a renal biopsy remains the cornerstone of diagnosis. Without histologic confirmation, clinical judgment alone would have favored immediate escalation to immunosuppressive regimens such as rituximab or cyclophosphamide, cyclophosphamide with glucocorticoid taper, or even consideration of plasma exchange in severe cases [4].

In our patient, the renal biopsy revealed acute tubular injury superimposed on chronic hypertensive nephrosclerosis, without histologic evidence of active ANCA-associated glomerulonephritis. By adopting a measured approach and proceeding with tissue confirmation, the real culprit behind the ongoing urinary abnormalities was identified [4].

The 2021 ACR/VF guidelines recommend tailoring therapy according to disease severity and organ-threatening involvement in ANCA-associated vasculitis. Complementing these recommendations, the 2024 KDIGO guidelines emphasize renal biopsy to confirm active glomerulonephritis before escalation of immunosuppressive therapies. The treatment of GPA is generally divided into induction of remission, maintenance, and prevention of relapse [4,12].

Initial therapy for GPA aims to induce remission using glucocorticoids along with either rituximab or cyclophosphamide, chosen based on disease severity. Plasma exchange may be an option for cases with life-threatening organ involvement. Maintenance is usually done with rituximab or azathioprine. With growing insight into complement-mediated pathophysiology, avacopan, which inhibits the C5a-C5aR1 axis, has also been widely incorporated into treatment guidelines in the induction phase for cases of renal involvement [1,4,9].

An additional unique aspect of this case is that the patient's symptoms have remained stable over the past two years with a combination of methotrexate and avacopan in the absence of ongoing glucocorticoids, a medication strategy not specifically addressed in current guideline recommendations [12].

In patients with non-severe or localized GPA, methotrexate has demonstrated comparable remission rates to cyclophosphamide at six months, though there is evidence of a high risk of relapse [13,14]. In another study analyzing the long-term follow-up of methotrexate, it showed less effective disease control than cyclophosphamide-based therapies [15]. Nonetheless, the recommendations favor the use of less toxic options before opting for cyclophosphamide or rituximab in refractory cases [12].

Avacopan, a complement C5a receptor inhibitor, when given along with standard induction therapy, can achieve better sustained remission at 52 weeks compared with the prednisolone taper strategy as described in one of the phase 3 randomized controlled trials, thereby paving the way for its incorporation into existing treatment guidelines worldwide as a steroid-sparing agent. However, it should be noted that the available evidence derives primarily from studies in moderate-to-severe ANCA-associated vasculitis with systemic involvement, and its role in isolated ENT-limited disease remains limited [4, 9].

Although the renal biopsy in our case confirmed the absence of systemic renal involvement, close surveillance for potential relapse remains warranted.

Published reports of nasal-limited GPA mention using methotrexate for non-severe cases, with an increase to rituximab for refractory cases. However, evidence describing the utilization of methotrexate and avacopan for ENT-restricted GPA is limited [11,16].

In our patient, a stable clinical course during follow-up was maintained for more than two years while receiving methotrexate and avacopan, a duration that is longer than typically reported with methotrexate monotherapy in published studies [13-15].

This case describes the clinical course of a patient with localized, non-organ-threatening GPA treated with this regimen. Longer-term follow-up data are currently limited at the time of writing; the patient remains under ongoing outpatient nephrology and rheumatology follow-up.

## Conclusions

This case explains the critical role of renal biopsy in assessing new urinary abnormalities in patients with localized GPA. The histopathology report prevented misclassification of renal involvement and avoided unnecessary changes to stronger immunosuppressive therapy. In addition, this case report provides insights into avacopan-methotrexate therapy in the management of localized GPA. Early recognition and personalized therapy remain the mainstay in attaining positive outcomes in localized presentations.

These findings highlight the importance of considering alternative renal pathologies in GPA patients who develop new urinary abnormalities and support the role of renal biopsy in guiding appropriate clinical decision-making before escalation of immunosuppressive therapy.
